# Design and characterization of a SYBR Green I-based melting curve method for investigation of HER2I655V polymorphism in breast cancer

**DOI:** 10.1186/s43141-020-00108-9

**Published:** 2021-01-11

**Authors:** Desriani, Azamris, Shabrina S. Ghaissani, Senja R. Kinanti, Muhammad A. Warisman, N. Fitria

**Affiliations:** 1 Research Center for Biotechnology, Indonesian Institute of Sciences (LIPI), Jl Raya Cibinong KM 46, Bogor, West Java Indonesia; 2 Division of Surgical Oncology Medical School of M. Djamil Hospital, Andalas University, Jl Perintis Kemerdekaan No. 94, Padang, West Sumatra Indonesia; 3 Department of Pharmacology and Clinical Pharmacy, Faculty of Pharmacy, Andalas University, West Sumatra, Indonesia

**Keywords:** Tm Shift SYBR Green I, qPCR assay, HER-2 Ile655Val Detection, Breast cancer, Trastuzumab

## Abstract

**Background:**

Breast cancer is a disease in which cell grows rapidly forming a mass in the breast. HER2 polymorphisms Ile655Val have been studied as biomarkers for breast cancer and may comprise a risk factor of cardiac toxicity for breast cancer-consuming trastuzumab. *Aim of work*: In this study, we developed a simple, low cost, and rapid test to detect polymorphism at HER2 gene using SYBR Green I-based melting curve method.

**Subjects and methods:**

In this report, we performed allelic discrimination with real-time temperature melting (Tm) Shift SYBR Green I-based melting curve method. The melting profiles of amplified DNA HER-2 Ile655Val and its characteristics were analyzed.

**Result:**

Tm value of HER2 GG and AA alleles were 85 ± 0.14 °C and 82.5 ± 0.23 °C, respectively, while cycle threshold (Ct) value of GG, AG, and AA alleles were 19.6 ± 0.27, 22.5 ± 0.23, 18.6 ± 0.22 correspondingly; furthermore, no template control has shown consisting Ct value at 31.18 ± 0.27. The developed methods’ characteristics were optimum annealing at 62 °C and Kappa coefficient value 1 with the mean almost consistent with PCR-sequencing. The coefficient of variability for intra-assay of GG, AG, and AA was in the range of 0.2–1%, while the coefficient of variability for inter-assay for each were in the range 0.7–1%. Further, based on PCR, shelf-life assay has shown stability for 3 months of storage observation.

**Conclusion:**

This approach may be considered as simple, rapid, and low cost supporting the rapid study of HER2 epidemiology. Furthermore, the developed methods potentially facilitate clinicians in dealing with breast cancer patients, especially in considering about the cardiotoxicity effect of trastuzumab.

## Background

According to the World Health Organization, breast cancer, commonly occurs in women with over 2 million new cases diagnosed in 2018 alone globally [1]. Approximately 30% of the total breast cancer sufferers are of type *HER-2*, which is an invasive and aggressive type of breast cancer. Human epidermal growth factor receptor2 (*HER-2*) is a proto-oncogene comprising of chromosomal 17q21 and encodes a transmembrane glycoprotein with tyrosine kinase activity. The polymorphism found in codon 655 (ATC/isoleucine to GTC/valine) in the transmembrane domain of the HER-2 protein is related to high risk of breast cancer [2, 3]. The previous study on the meta-analysis of *HER-2* Ile655Val polymorphism stated that it significantly contributes to the risk of breast cancer risk [4, 5]. The existence of valine in the transmembrane domain tends to affect the stability of the receptor active state, thereby decreasing the speed of endocytosis and accelerating the receptor recycling, which leads to the formation of breast cancer. However, this is contrary with isoleucine which destabilizes the formation of HER-2 heterodimers [6]. Furthermore, HER2-expressing cells acquired the characteristics of tumor cells [7]. A recent study on 4167 Shanghai patients showed high levels of *HER-2* Ile655Val polymorphism present in breast cancer phenotypes in the entire population. Treatment with trastuzumab a monoclonal antibody that specifically binds to HER2 disrupts the downstream pathways of HER-2 Ile655Val polymorphism [8]. Similarly, the presence of Val allele associated with cardiomyocytes was also reported to be highly sensitive to trastuzumab [7, 9, 10]. The distribution of *HER-2* polymorphism showed variations in frequency of Val/Val and Ile/Val genotype in different ethnicities such as Caucasian (5.4%, 29.2%), African–Americans (5.4%, 38.9%), Saudi Arabians (2.0%, 17.8%), Chinese (0.3%, 21.7%), and Filipinos (15%, 1.3%). Meanwhile, in African populations, the Val/Val genotype was the same except in Kenya and Sudan with Ile/Val percentages of 26% and 17.3%, respectively [11]. Understanding disease-related single-nucleotide polymorphisms (SNPs) will facilitate *HER-2* Ile655Val polymorphism genotyping study.

Most of the studies on *HER-2* Ile655Val polymorphism detection were carried out using the PCR-restriction fragment length polymorphism (RFLP) and TaqMan genotyping method [2, 7, 12, 13]. Each has advantages and disadvantages; for instance, the PCR-RFLP is not of high-throughput detection with post-PCR confirmation, while TaqMan is used to detect high throughput in real-time [13–19]. Here, we performed allelic discrimination with temperature melting (Tm) Shift SYBR Green I methods using two forward primers specific to the targeted allele and one typical reverse primer. The melting temperature was investigated in order to discriminate the alleles.

## Subjects and methods

This study was carried out at Molecular Biology and Diagnostic Laboratory LIPI, Cibinong Science Centre, Indonesia.

### Subject

Tumor tissue samples were taken from 30 patients (minimal samples) with primary breast cancer by biopsy from several hospitals in the province of West Sumatra, Indonesia. This research had acquired an ethical approval from the Indonesian Ministry of Health and informed consent from patients. The obtained samples were stored at − 20 °C with genomic DNA extracted from the frozen breast cancer tissues using Purelink from Invitrogen. Furthermore, the DNA concentrations were determined by measuring the absorbance rate using a spectrophotometer at 260/280 nm. All of the 30 samples were already checked for its sequence.

### Methods

#### Quantitative real-time PCR

Single-nucleotide polymorphisms (SNPs) play an important role in determining various cancer types and are capable of serving as diagnostic for its treatment. This research utilized two forward primers for each SNP with the first comprising three mismatched bases from 3′ with long GC tails added at 5′ end. Meanwhile, the second is a forward primer that has no mismatch with a short GC tail located at the same position as previous forward primers. A standard reverse primer was designed for both alleles: Forward1: (G allele) 5′GCGGGCAGGGCGGCCCAGCCCTCTGACGTCCAGCG 3′. Forward 2: (A allele) 5′GCGGGCCCAGCCCTCTGACGTCCATCA 3′. Reverse: 5′CACCCCCAAGACCACGACCA3′ as illustrated in ref [14, 19]. The study optimized qPCR primer proportion formulation and an annealing temperature of 60–62 °C. Furthermore, qPCR was conducted in 10 μl volumes with the amplification mixture for each reaction comprising of 3.4 μl PCR Grade Water Thermoscientific, 5 μL 2x KAPA SYBR^TM^ Fast Green I, 0.175 Forward Primer *HER-2* gene 1, 0.2 μl Forward primer *HER-2* gene 2, 0.3 μl Reverse primer *HER-2* gene, and 1 μl DNA template. The temperature cycling process was carried out at 95 °C for 3 s, with 35 cycles of denaturation process at 95 °C for 10 s, annealing at 62 °C for 30 s, extension at 72 °C for 30 s, and a final extension at 72 °C for 30s. Furthermore, the melt curve and peak analyses were carried out immediately at a melting rate value of 0.2 °C/min, from 65 to 95 °C. A graph of − dF/dT against T formula (F is fluorescence, T is temperature) was plotted to determine the melting peaks.

#### PCR reagent shelf-life assay

Components of PCR regen prepared in shelf assay include Forward GG Primer, Forward AA Primer, Reverse Primer, 2x KAPA SYBR^TM^ Fast Green I, and PCR Grade Water Thermoscientific Nuclease Free. The PCR regen was stored at − 20 °C, 4 °C, and room temperature. Subsequently, the melt peak assay was used to examine the stability value for each reaction daily, weekly, and monthly for 3 months.

#### Data analysis

The developed methods were examined based on the melt curve and peak analyses. In addition, the repeatability and reproducibility processes were analyzed using inter- and intra-run variability assays supported with Microsoft excel 2003. Meanwhile, the PCR set analysis was performed using SPSS Cohen’s Kappa.

## Result

### Optimization of SYBR Green I for HER-2 SNP detection

A total of two forward primers were used for the optimization of SYBR Green I-based melting curve method, which is specific to the targeted allele and one standard reverse primer. Furthermore, the primer design was carried out by introducing SNP at the 3′ end and a mismatch at the third bases of the forward primer, with the addition of varying lengths of GC tail.

**Fig. 1:**
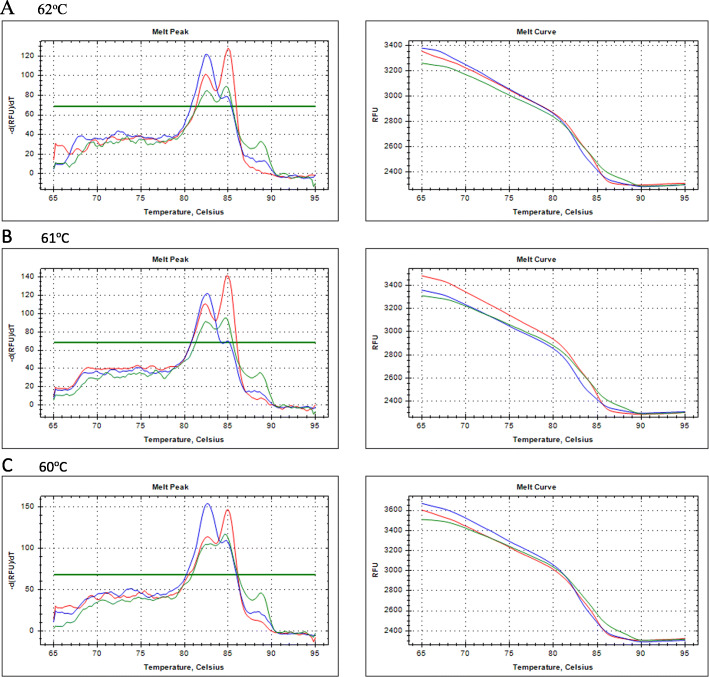
Melting curve and melting peak at 62 °C, 61 °C, and 60 °C annealing temperature (AA: blue, AG: green, GG: red)

This research previously optimized the primary proportion to differentiate SNPs and non-SNPs. There were variation A (0.175 μM Fw GG, 0.2 μM Fw AA, and 0.3 μM rev), variation B (0.2 μM Fw GG, 0.2 μM Fw AA, and 0.3 μM rev), variation C (0.3 μM Fw GG, 0.2 μM Fw AA, and 0.2 μM rev), variation D (0.35 μM Fw GG, 0.2 μM Fw AA, and 0.2 μM rev), variation E (0.4 μM Fw GG, 0.2 μM Fw AA, and 0.2 μM rev), and variation F (0.5 μM Fw GG, 0.2 μM Fw AA, and 0.2 μM rev). From those six, variation A has shown the best proportion to differentiate SNPs and non-SNPs. With more concentration of Fw GG, the melt curve will be able to strongly recognize G allele; this effected especially to GG and AG alleles which cannot be differentiated. Furthermore, the best formulation was used to optimize the annealing temperature at 62 °C with a sharper melt curve and peak value, as shown in Fig. 1.

**Fig. 2:**
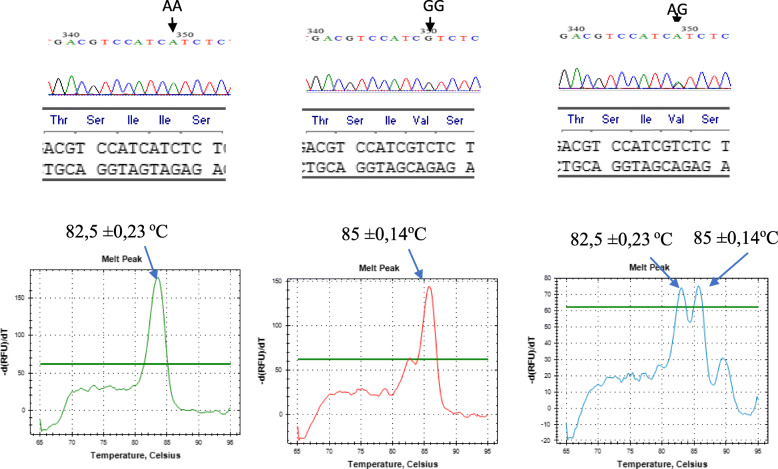
SYBR Green I-based real-time PCR analysis for the detection of SNP and non-SNP HER-2 Ille655Val

The methods were repeated with optimum primer proportion and optimum annealing temperature to confirm the consistency of the melting curve and melting peak. The results showed that the created mismatch primers were able to discriminate SNP from non-SNP consistently. Tm value for SNP HER-2 GG and WT (AA) were 85 ± 0.14 °C, and 82.5 ± 0.23, while cycle threshold (Ct) value for GG, AG, and AA were 19.6 ± 0.27, 22.5 ± 0.23, and 18.6 ± 0.22, respectively. The no template control (NTC) consists of Ct value at 31.18 ± 0.27 °C with a distance temperature of 11 °C from the target. Non-specific melt peak appears at a temperature of 90 °C melting upon AG, and slightly on GG, with a melting temperature similar to AA, as shown in Fig. 2.

### Repeatability and reproducibility assay

The reproducibility of the assay was resolute by testing triplicate using inter- and intra-assay comparison, conducted for 3 days. Furthermore, the coefficient of variability for the intra-assay and inter-assay of GG, AG, and AA was in the range of 0.2–1% and 0.7–1%, respectively, as shown in Table 1. These data indicated that the assay was repeatable and highly reproductive, with no pipetting error and low variation.

**Table 1:** Repeatability and reproducibility of *HER-2* Ille655Val real-time assay

Genotype	Intra-run assay (Ct)						Inter-run assay (Ct)
	Day 1		Day 2		Day 3		CV
	SD	CV	SD	CV	SD	CV	
AA	0.22	1.17	0.17	0.908	0.043	0.22	0.77
AG	0.23	1.041	0.136	0.6	0.06	0.26	0.78
GG	0.26	1.32	0.122	0.62	0.125	0.65	1.09

*SD* standard deviation, *CV* coefficient of variability

### Assay performance on the clinical sample analyzed with Kappa Cohen

Based on the comparison and direct sequencing results of the developed method with Kappa statistics, a kappa value of 1 was obtained, as shown in Table 2.

**Table 2:** Results of Tm Shift SYBR Green I and PCR-sequencing of 30 samples

No of samples	Test result	
	Tm Shift SYBR Green I	PCR-sequencing
22 samples	AA	AA
7 samples	AG	AG
1 sample	GG	GG

### Shelf-life assay

**Fig. 3:**
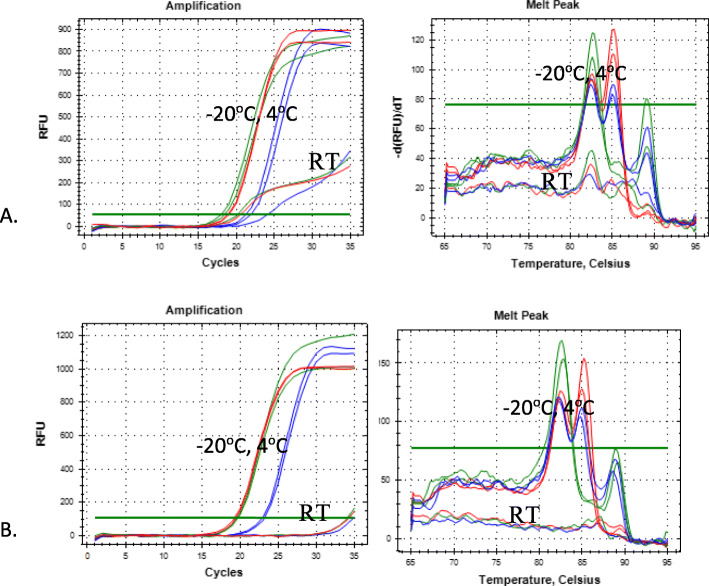
Shelf-life assay of chemical used for SYBR Green I for *HER-2* SNP detection during storage at room temperature (RT), 4 °C and − 20 °C. **a** Three-week storages. **b** Four-week storages

Shelf-life test was conducted by observing the consistency of *HER-2* SNP melting peak values at chemical regen storage temperatures of − 20 °C, 4 °C, and room temperature for 3 months. All chemical reagents were protected from light, and the tests were investigated five times daily, for 4 days in a week and for 3 months. The result showed that the chemical regent storage at − 20 °C and 4 °C had consistent melt curve and melt peak values for 3 months, while at room temperature it had low and loose genotyping ability after storing for 3 weeks. Figure 3 shows only 3 and 4 weeks of observation, because periods at − 20 °C and 4 °C had a similar melt curve pattern.

## Discussion

This study developed *HER-2* Ile655Val polymorphism genotyping using qPCR with SYBR Green I as the fluorescence dye. The main advantage of SYBR Green I compared with other real-time dyes is that it is easy to use, cost-effective, and rapid. The amplification for specific DNA targets associated with SYBR Green I binding and its analysis does not use an additional step, with agarose electrophoresis, as the endpoint PCR. Liu et al. and Wang et al. studies were referenced in this study for the development of various genotyping methods. According to Liu et al., an additional mismatched site on the second, third, and fourth sites from 3′ reported different effects on SNP discrimination. The primers’ design had an additional mismatched in the third nucleotide position from the 3′-end, with the highest allele specificity [20].

Furthermore, Wang et al. reported that additional GC tails at the 5′ orientation to forward primer which consists of 14 bp and 6 bp, respectively, for each forward primer increased the method’s discrimination ability and improved the throughput [15]. Based on those two considerations, SNP (GG and AG alleles) and non-SNP (AA allele) HER-2 PCR products are distinguished for each assay in single closed tubes, which eliminates the risk of PCR contamination. In the preliminary study titled “Developed *HER-2* I655V detection based on SYBR Green I-based melting curve method,” it was shown that an un-sharp melt curve target and a clearly additional curve appear at temperatures of 75 °C. Additional curve at 75 °C indicates the existence of a dimer primer which means low specificity [21–24]. To develop a real-time PCR research validation, the specificity, repeatability, reproducibility, and stability parameters of the assays were considered [25, 26].

The annealing temperature is one of the critical factors that affected the primer specificity. However, this study utilized an optimum annealing temperature of 62 °C because the melt curve and melt peak analysis showed sharper and firm values at this point. Each allele, AA, AG, and GG, have specific melt peak patterns and are capable of discriminating against each other. Although the unspecified product that appeared in the AG allele was detected at 90 °C, they were ignored due to different melting temperatures with the targets of 85 ± 0.14 °C for G and 82.5 ± 0.23 for A. Non-specific products also slightly appeared on GG allele detection, with differing melt peaks similar to the AA allele melting temperature. Those unspecific peaks were also ignored because the proportion of the melting peak for the GG allele was consistently higher than the non-specific melting peak. This is different from the GG melt peak pattern, where AG was formed at the same level for both A and G alleles.

Several statistic studies have been carried out to test the accuracy and performance of the diagnostic or detection kit. For the methods’ development accuracy, the developed methods were compared with a standard gold method of PCR-sequencing using 30 frozen breast cancer tissue samples as DNA genomic sources. Based on a statistic study with Kappa Cohen’s analysis, the results meet the requirement by producing one kappa value which means that it is in almost perfect agreement.

Furthermore, the coefficient of variability for intra-assay and inter-assay of GG, AG, and AA were in the range of 0.2–1 and 0.7–1%. These values showed that the acceptable max value is less than 10 and 15%, respectively. This value provides no pipetting error, and the developed methods have high reproducibility [27, 28]. It also reflects the excellent performance of developed qPCR methods [25, 26] while the shelf life of used chemical reagent was stable during the 3-month storage at − 20 °C and 4 °C as shown by the consistent melt curve and melt peak results. In addition, since all reactions are performed and detected in one closed single tube, this eliminates the risk of PCR contamination as another advantage of the developed method.

## Conclusions

In conclusion, this study developed a simple and low-cost SYBR Green I-based melting curve method for the rapid detection of HER2 polymorphism. The Tm Shift SYBR Green I-based melting curve method was used to determine the allelic discrimination in real-time followed by the analyses of the melting profiles of amplified DNA *HER-2* Ile655Val and its characteristics. The real-time PCR was able to discriminate SNP (GG or AG allele) from non-SNP (AA allele). Furthermore, the Tm value for HER-2 GG and AA alleles were 85 ± 0.14 °C and 82.5 ± 0.23 °C, while Ct value for GG, AG, and AA alleles were 19.6 ± 0.27, 22.5 ± 0.23, and 18.6 ± 0.22, respectively. The NTC comprises of Ct value at 31.18 ± 0.27. The developed methods were obtained at an optimum annealing temperature of 62 °C and Kappa coefficient value of 1, with the mean in accordance with PCR-sequencing. Meanwhile, the coefficient of variability for intra- and inter-assays of GG, AG, and AA were in the ranges of 0.2–1% and 0.7–1%, respectively, with a 3-month stable PCR shelf-life assay stored for observation. These data designated that the assay was repeatable and highly reproducible, with no pipetting error and low variation. Furthermore, the PCR regen shelf-live assay showed stability for 3 months of storage observation. Real-time PCR using SYBR green I Tm Shift for *HER-2* SNP Ile655Val was proven as a simple, efficient, specific, and reproducible method. Therefore, this approach might be considered effective to detect HER2-I655V polymorphism in patients with breast cancer, as it could also facilitate the rapid study of HER-2 epidemiology.

## Data Availability

All data generated or analyzed during this activity are included in this published article.

## Abbreviations

Tm: Temperature melting HER-2 Human epidermal growth factor receptor 2 Ile655Val Isoleusin655Valine qPCR Quantitative polymerase chain reaction RFLP Restriction fragment length polymorphism SNP Single-nucleotide polymorphism Ct Cycle threshold NTC No template control
